# Regulation of a phage defence island by RptR, a novel repressor that controls restriction–modification systems in diverse bacteria

**DOI:** 10.1093/nar/gkaf645

**Published:** 2025-07-12

**Authors:** YuGeng Zhang, Marion Schuller, Ivan Ahel, Tim R Blower, Rachel M Exley, Christoph M Tang

**Affiliations:** Sir William Dunn School of Pathology, University of Oxford, South Parks Road, Oxford OX1 3RE, United Kingdom; Sir William Dunn School of Pathology, University of Oxford, South Parks Road, Oxford OX1 3RE, United Kingdom; Sir William Dunn School of Pathology, University of Oxford, South Parks Road, Oxford OX1 3RE, United Kingdom; Department of Biosciences, Durham University, South Road, Durham DH1 3LE, United Kingdom; New England Biolabs, 240 County Road, Ipswich, MA 01938, United States; Sir William Dunn School of Pathology, University of Oxford, South Parks Road, Oxford OX1 3RE, United Kingdom; Sir William Dunn School of Pathology, University of Oxford, South Parks Road, Oxford OX1 3RE, United Kingdom

## Abstract

Bacteria encode a panoply of defence systems to overcome phage infection. In recent years, over 100 defence systems have been identified, with the majority of these found co-localized in defence islands. Although there has been much progress in understanding the mechanisms of anti-phage defence employed by bacteria, far less is known about their regulation before and during phage infection. Here, we describe RptR (RMS-proximal transcriptional regulator), a small transcriptional regulator of a defence island in enteropathogenic *Escherichia coli* composed of a toxin–antitoxin system, DarTG2, embedded within a Type I restriction–modification system (RMS). We determined the molecular structure of a RptR homodimer and, using transcriptional reporter and *in vitro* DNA binding assays, show that RptR represses the promoter of the defence island by binding to a series of three direct repeats in the promoter. Furthermore, we demonstrate, using the structural models of RptR validated with electrophoretic mobility shift assays, that the minimal RptR binding site is a 6-bp palindrome, TAGCTA. Both RptR and its binding site are highly conserved across diverse bacterial genomes with a strong genetic association with Type I RMS, highlighting the role of RptR as a novel regulatory component of an important mechanism for anti-phage defence in bacteria.

## Graphical Abstract

**Graphical Abstract:**
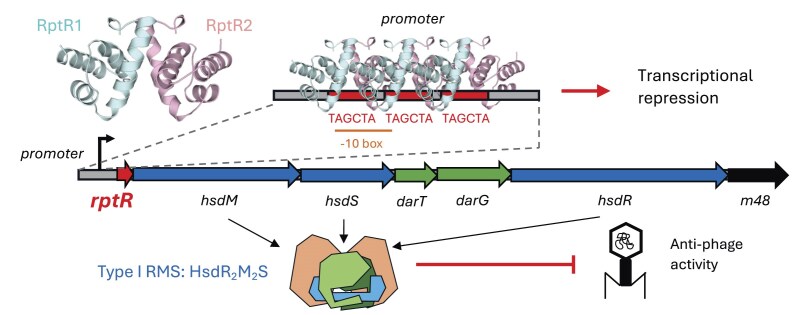

## Introduction

Phages and bacteria have been engaged in co-evolutionary competition for eons. While a successful virion must infect and propagate within a bacterial host, the survival of bacteria is dependent on the success of a diverse array of anti-phage defence systems. In recent years, the availability of whole genome sequences and computational predictions of gene function(s) have accelerated the discovery of over 100 novel defence systems, with the biochemical activity of many elucidated [1–4].

Among the diverse defence systems found in bacteria, restriction–modification systems (RMSs) are the most prevalent and present in over 80% of sequenced prokaryotic genomes [5]. RMSs selectively degrade unmethylated phage DNA but not methylated host DNA [6, 7]. The general principle of host DNA methylation and degradation of unmethylated foreign DNA is employed by Type I–III RMSs, while Type IV enzymes degrade modified DNA [8]. Type I RMSs include a methyltransferase complex, containing HsdM methyltransferase and HsdS specificity subunits in an HsdM_2_S stoichiometry, that methylates *N*_6_-adenine on hemi-methylated host DNA. Upon recognition of unmethylated DNA motifs, a cleavage-competent HsdR_2_M_2_S_1_complex, containing two HsdR endonuclease subunits, catalyses DNA translocation, looping, and cleavage [9, 10]. This mechanism contrasts with, for instance, the majority of Type II RMSs where methylation and endonuclease activities are performed by separate enzymes that do not interact directly [11].

RMSs are canonical mediators of non-abortive anti-phage defence systems as they do not compromise host cell growth or viability following activation. Conversely, abortive infection (Abi) mechanisms enable protection at the level of a bacterial population by ensuring that no phages are produced by infected cells that fail to replicate or die [12, 13]. Toxin–antitoxin (TA) modules include a highly diverse group of Abi systems composed of a stable toxin that is neutralized by a labile antitoxin in the absence of phage infection. Upon phage infection and translational inhibition, antitoxin levels decrease, leaving sufficient free toxin to prevent phage replication by inducing cell dormancy or death. The DarTG TA system mediates Abi and is categorized into two families, DarTG1 and DarTG2. Both families carry the DarT toxin that ADP-ribosylates primarily single-stranded DNA at either guanine (DarTG1) [14] or thymidine bases (DarTG2) [15, 16], with the latter leading to replication fork stalling and host cell death [17]. DarT-mediated ADP-ribosylation is reversed by the activity of the ADP-ribosyl hydrolase antitoxin, DarG, which in the case of DarG2 also inactivates DarT via physical sequestration [15]. Members of both DarTG1 and DarTG2 families can provide robust anti-phage defence when introduced into *Escherichia coli* [18].

Although defence systems have usually been characterized in isolation, many prokaryotic genomes encode multiple anti-phage defence systems [5]. Genes encoding ∼70% of defence systems are co-localized within defence islands [19], with several examples of genes of one defence system embedded between genes of another system (e.g. Septu in Hma systems) [20, 21]. This genetic clustering of anti-phage defence systems has enabled the identification of candidate bacterial defence modules through ‘guilt-by-association’ and ‘guilt-by-embedding’ [1, 19, 20]. The presence of multiple distinct defence systems can offer broad protection against diverse phages, with combinations of only two discrete systems providing complementary and/or synergistic protection against phage [22, 23]. Furthermore, multiple defence systems are often located within, or adjacent to, mobile genetic elements, enabling transfer within and across bacterial species.

The presence of multiple, potentially toxic, defence systems within bacteria raises the question of how such defence systems are regulated. For this, bacteria must not only time the activation of defence systems to coincide with phage infection but also strictly control defence systems to prevent toxicity due to inappropriate expression of effectors in the absence of infection. For instance, the CapRel [24] and CmdTAC [25] systems remain inactive until phage capsid proteins are detected, while the PARIS, Gabija, and Zorya Type II systems are activated upon sensing the T7 phage-encoded anti-restriction protein, Ocr [3, 22]. In the absence of phage infection, defence systems are often repressed. For example, Type I RMSs can be cleaved by host proteases, such as ClpX/P, to degrade the endonuclease subunit upon loss of DNA hemi-methylation, following DNA damage or introduction of the system into a new host [26, 27]; this process is referred to as restriction alleviation. TA systems, meanwhile, can be controlled by autoregulatory feedback loops with the DNA-binding domains of antitoxins binding to and repressing their native promoter [28].

Aside from autoregulation, some transcriptional regulators are expressed as a part of anti-phage defence systems. The controller (C) proteins of Type II RMSs, encoded immediately upstream of genes for restriction endonucleases (REases), promote expression of the endonuclease at low concentrations while repressing it at high concentrations, thereby keeping REase expression within tightly defined limits [29, 30]. Meanwhile, the WYL helix–turn–helix (HTH) transcriptional repressors, BrxR/CapW [31, 32] and CapR [33], specifically control expression of the BREX and CBASS defence systems. Although they are frequently associated with these defence systems, *brxR*/*capW* and *capR* are only found in a relatively small proportion of bacterial genomes and thus represent the tip of the iceberg of defence system regulators.

Here, we identified RptR (RMS-proximal transcriptional regulator), a novel, small transcriptional repressor of a seven ORF (open reading frame) defence island in enteropathogenic *E. coli* (EPEC) encoding a DarTG2 system embedded in a Type I RMS. We show that this defence island is active against selected phages from the Durham collection [34] with transcription of *hsdM* upregulated within 1 h of infection. We present the crystal structure of RptR that is encoded by the first ORF of the defence island and forms a homodimer of two alpha-helical subunits. RptR represses its own promoter by binding to a series of three 6-bp palindromic motifs overlapping the −10 box. Despite the presence of three binding sites, only two intact RptR binding motifs are required for high-level binding and transcriptional repression. Bioinformatic analyses of RptR homologues across a set of diverse bacterial genomes reveal that RptR and its binding motif are highly conserved in *Gammaproteobacteria*. In the genomes analysed, RptR is frequently found upstream of a Type I RMS, demonstrating that RptR provides a novel way of regulating the expression of these important and prevalent defence systems.

## Materials and methods

### Bacterial strains, culture conditions, and reagents

Bacterial strains and plasmids used in this study are listed in Supplementary Table S1. Phage isolates used in this study are listed in Supplementary Table S2. Unless otherwise stated, bacteria were grown in lysogeny broth (LB, 20 g/l) at 37°C with shaking (180 rpm). Solid growth media were prepared by adding 1.5% agar (w/v). Mid-log phase bacterial cultures were prepared by diluting overnight cultures (1:1000, v/v), and then allowing growth to an optical density (OD_600_) of 0.5–0.7. Growth media were supplemented with antibiotics at the following concentrations: ampicillin, 100 μg/ml; chloramphenicol, 20 μg/ml; and kanamycin, 50 μg/ml.

### Plasmid construction

Plasmids were constructed by Gibson assembly (NEBuilder^®^HiFi DNA Assembly Master Mix, NEB) of PCR (polymerase chain reaction)-amplified vectors and inserts; primers are listed in Supplementary Table S3. Plasmids were introduced into *E. coli* DH5α by heat shock [35]. pBR322-RRDM, carrying the entire RptR-RMS-DarTG-M48 defence island with its promoter (i.e. 159 bp upstream of *rptR*), was assembled from pBR322 (primers YZ29/YZ30) and a 9.8-kb PCR product containing the defence island from *E. coli* O127:H6 E2348/69 (EPEC) (NC_011601) (primers YZ31/YZ127). pBR322-GFP_pN-T7_RBS_ was assembled from pBR322 with the *rptR* promoter (primers YZ30/YZ160) and an insert containing the ORF encoding superfolder GFP [36] and the T7 gene 10 extended ribosome-binding site [37] (primers YZ158/YZ159), amplified from pCONJ5HP (Tang group). GFP reporter plasmids containing the *rptR* promoter with various combinations of direct repeats (DRs) were derived from a linearized, promoterless pBR322-GFP_pN-T7_RBS_ (primers YZ29/YZ303) and synthesized promoter fragments (Integrated DNA Technologies). For arabinose-inducible expression of His_6_-RptR, a fragment encoding RptR with a primer-encoded N-terminal hexa-histidine (His_6_) tag (primers YZ202/YZ203) was amplified from EPEC gDNA and assembled with pBAD33 (Addgene), generating pBAD33-His_6_-RptR. Similarly, pET28a-His_6_-3C-RptR was assembled from pET28a (primers YZ221/YZ172), containing an N-terminal His_6_ tag and a 3C protease cleavage site, with the *rptR* ORF (primers YZ222/YZ223). Site-directed mutagenesis to introduce S60G and L74R substitutions in RptR was performed with primers containing a 5′ overlap to the sequence adjacent to the modified site (pBAD33: S60G—primers YZ204/YZ111, L74R—primers YZ206/YZ202; pET28a: S60G—primers YZ204/YZ94). Fragments were assembled with their respective backbone fragments to form a complete plasmid (pBAD33: S60G—primers YZ205/YZ112, L74R—primers YZ201/YZ182; pET28a: S60G—primers YZ205/YZ96). Plasmids were purified with the GenElute™ Kit (Merck) and verified by sequencing (Plasmidsaurus Inc.).

### Phage propagation and plaque assay

Phages were propagated by adding 10-fold serial dilutions of phage stocks at a 1:400 (v/v) ratio into 4 ml of 0.35% top agar containing 100 μl of an overnight culture of *E. coli* DH5α. Top agar was poured on a 1.5% agar base and incubated for 6 h at 37°C, and then overnight at room temperature (RT). Top agar of plates with confluent lysis was collected into 30-ml glass tubes containing 3 ml of buffer (10 mM Tris–HCl, pH 7.4, 10 mM MgSO_4_, 0.1% porcine gelatin). The suspension was vortexed for 2 min and incubated at 4°C for 30 min. The phage-enriched liquid fraction was obtained by centrifugation (4300 *× g*, 20 min, 4°C) and stored at 4°C with chloroform (1:30, v/v).

Plaque assays were performed by spotting 10-fold serial dilutions of lysate onto top agar containing 500 μl *E. coli* overnight cultures. Plates were incubated at 37°C for 6 h prior to enumeration of plaques.

### Plaque assay with RptR induction

*Escherichia coli* DH5α carrying pBR322-RRDM and pBAD33-His_6_-RptR (WT/S60G) or empty pBAD33 vector was grown overnight with either 0.2% arabinose or 0.8% glucose to induce or repress RptR expression, respectively. Plaque assays were performed as described above with either 0.2% arabinose or 0.8% glucose added to both agar layers. Plaques were enumerated after overnight incubation at 37°C.

### GFP reporter assay

Mid-log phase *E. coli* MG1655, carrying a dual-plasmid reporter system (pBR322-GFP and pBAD33-His_6_-RptR or empty pBAD33), was grown in LB with 0.8% glucose (w/v). Bacteria (1 ml, OD_600_ ∼ 0.5) were pelleted and resuspended in 1 ml of LB containing 0.2% arabinose (w/v). Cells were diluted to an OD_600_= 0.01 in LB with arabinose in 96-well plates (Thermo Scientific). The OD_600_ and fluorescence at 495 nm were measured over 16 h in a FLUOstar Omega platereader (BMG Labtech) with incubation at 37°C and 200 rpm.

For expression of RptR, *E. coli* MG1655 carrying the dual-plasmid reporter system was resuspended in LB with glucose as above. Cells were then diluted in media lacking glucose to an OD_600_= 0.01 and added to 96-well plates (Thermo Scientific). After 3 h, arabinose was added to a final concentration of 0.2% (w/v). Cell density/GFP fluorescence was measured as above.

### Protein purification

Recombinant RptR with an N-terminal His_6_ tag and 3C protease cleavage site was expressed in *E. coli* BL21 (DE3) carrying pET28a-His_6_-3C-RptR or pET28a-His_6_-3C-RptR_S60G_. Protein expression was induced by addition of 0.5 mM isopropyl β-D-1-thiogalactopyranoside (IPTG) at mid-log phase for 16 h at 18°C. Bacterial pellets were resuspended in 20 ml lysis buffer [50 mM Tris–HCl, pH 8, 500 mM NaCl, 0.5% Triton X-100, 10% (v/v) glycerol, 100 μg/ml lysozyme, 1 μg/ml DNase I, and 1× cOmplete EDTA-free Protease Inhibitor cocktail (Roche) with 1 mM tris(2-carboxyethyl)phosphine and 20 mM imidazole], and then lysed by sonication (Soniprep 150, MSE Supplies LLC). Lysates were loaded onto a gravity-flow chromatography cartridge containing Ni-NTA FF crude agarose resin (Cytiva), pre-equilibrated with five column volumes (CV) of equilibration buffer (50 mM Tris–HCl, pH 8, 500 mM NaCl, 10% glycerol, 1% Triton X-100, and 20 mM imidazole). The protein was washed with 20 CV of wash buffer (50 mM Tris–HCl, pH 8, 500 mM NaCl, 5% glycerol, and 20 mM imidazole) and eluted with elution buffer (50 mM Tris–HCl, pH 8, 500 mM NaCl, 5% glycerol, and 500 mM imidazole).

Relevant fractions were pooled and concentrated using a Pierce™ Protein Concentrator (PES, 3K MWCO, 2–6 ml). Purified protein was obtained by subsequent size-exclusion chromatography (SEC, Superdex™ 75 Increase 10/300 GL, Cytiva) in SEC buffer (25 mM Tris–HCl, pH 8, 200 mM KCl) using an ÄKTA pure™ system (Cytiva). Protein aliquots were frozen in storage buffer (25 mM Tris–HCl, pH 8, 200 mM KCl, 10% glycerol) at −70°C. Proteins were separated on SDS–PAGE (sodium dodecyl sulphate–polyacrylamide gel electrophoresis) gels (4% stacking/20% resolving) followed by Coomassie blue staining. For western blots, SDS–PAGE-separated proteins were transferred onto Protran™ 0.2-μm nitrocellulose membranes (Cytiva) and probed with an anti-6X His tag^®^ primary monoclonal antibody (Abcam, ab18184; 1:5000, v/v), followed by a horseradish peroxidase-conjugated anti-mouse IgG secondary polyclonal antibody (Dako, P0447; 1:10 000, v/v), and visualized using the Amersham ECL detection reagent (Cytiva).

### Protein crystallization and structure determination

His_6_-3C-RptR was purified as above and concentrated to 12.6 mg/ml, as measured using a NanoDrop™ 2000 spectrophotometer (Thermo Scientific). Crystallization trials were set up across gradients of potassium thiocyanate (25–200 mM) and PEG 2000 MME (24%–35%) using a dragonfly^®^ crystal pipetting robot (SPT Labtech). One hundred and fifty nanolitre drops containing either a 1:1 or 1:2 volumetric ratio of protein to crystallization solution were generated using a mosquito^®^ pipetting robot (SPT Labtech). Protein crystals were grown at RT for 7 days using the sitting-drop vapour diffusion method with a 1:2 volumetric ratio of protein to crystallization solution (150 mM potassium thiocyanate, 32% PEG 2000 MME). Reservoir solution (70 μl) was compositionally identical to the crystallization solution. Crystals were cryo-protected in reservoir solution containing 20% ethylene glycol and immediately flash-frozen in liquid nitrogen. X-ray diffraction data were collected on the I04 beamline at Diamond Light Source (Oxfordshire, UK), and initial data processing performed using autoPROC [38].

The crystal structure of RptR was solved by molecular replacement with an AlphaFold 3 model of dimeric RptR using the Phaser-2.5.0 program and initially refined with Refmac5 [39], both from within the CCP4 suite [40, 41]. Per residue rigid-body and rotamer refinement was performed in Coot [42] and final refinement performed using the phenix-refine tool within the PHENIX suite [43]. The final structure was visualized in PyMOL (PyMOL Molecular Graphics System, Version 3.10, Schrödinger, LLC). Hydrophobicity plots were generated in PyMOL using the color_h script (https://pymolwiki.org/index.php/Color_h) employing the Eisenberg hydrophobicity scale [44].

### Estimation of His_6_-3C-RptR molecular weight by analytical SEC

Gel filtration low molecular weight (MW) calibration standards (Cytiva) were mixed to a final concentration with 3 mg/ml of each of the following proteins in SEC dilution buffer (Tris–HCl, pH 7, 200 mM NaCl): conalbumin (CON), ovalbumin (OVA), carbonic anhydrase (CAR), ribonuclease A (RIB), aprotinin (APR). A 200 μl volume of mixed standards was injected into an ÄKTA pure™ system (Cytiva) and separated by SEC on a Superdex™ 75 Increase 10/300 GL column (Cytiva). The void volume (*V*_o_) was determined as the elution volume of Blue Dextran 2000 (Cytiva). Normalized elution volumes (*K*_av_) were calculated using the following relationship, where *V*_e_ denotes the elution volume and *V*_c_ denotes the total column volume:


\begin{eqnarray*}
{{K}_{{\rm av}}} = \ \frac{{{{V}_{\rm e}} - \ {{V}_{\rm o}}}}{{{{V}_{\rm c}} - {{V}_{\rm o}}}}.
\end{eqnarray*}

A standard curve was fitted to a plot of *K*_av_ against log_10_(MW (kDa)) in GraphPad Prism and the estimated MW of His_6_-3C-RptR interpolated using elution volumes determined during protein purification.

### Electrophoretic mobility shift assay

Unlabelled double-stranded DNA (dsDNA) probes were prepared by hybridizing 60-bp primers (50 μM each; Merck) containing *rptR* promoter direct repeat (DR) sequences (Supplementary Table S3). Briefly, complementary primers were incubated at 94°C for 3 min in buffer (30 mM Tris–HCl, pH 8, 3 mM EDTA, pH 8, 150 mM NaCl) and cooled to 55°C in a heat block. Protein–DNA binding was assessed in 20 μl of EMSA (electrophoretic mobility shift assay) buffer (25 mM Tris–HCl, pH 8, 150 mM NaCl, 5 mM MgCl_2_) containing 0.05 μM dsDNA and 0, 0.6, or 1.2 μM RptR. For RptR titration curves, EMSAs were set up as above with concentrations of RptR ranging from 0.025 to 1.2 μM. Reactions were incubated at 37°C for 30 min, and then cooled to 4°C. Native agarose gel loading dye (Invitrogen) was added to reactions prior to separation on 8% TBE–PAGE gels (120 V, 30 min, 4°C) in 0.5× TBE running buffer. Bands were visualized with a UV transillumination in a Gel Doc™ XR+ imager after staining with SYBR™ Gold.

RptR titration curves for estimating binding parameters were obtained by quantification of free DNA bands in ImageJ at each RptR concentration. Values for free DNA were subtracted from, and then normalized to, levels at 0 μM RptR to give the percentage of bound DNA. The binding curve was fitted and binding parameters estimated using the specific binding curve model in GraphPad Prism.

### Gene expression analysis

Mid-log *E. coli* DH5α were diluted to an OD_600_= 0.1 in 10 ml LB. Cultures were inoculated with Trib phage at a multiplicity of infection (MOI) of 100 with one culture flask inoculated with the same volume of phage buffer as a control. Flasks were incubated at RT for 5 min to allow phage adsorption and then transferred to a 37°C shaking incubator (135 rpm). At time points post-infection, 1 ml samples were collected and bacteria were pelleted (21 000 × *g*, 1 min, 4°C). Phage infection was arrested by flash-freezing cell pellets in liquid nitrogen for 2 min.

For RNA extraction, cell pellets were transferred into 2-ml Safe-Lock tubes (Eppendorf) and resuspended in 200 μl TRIzol™ Max™ Bacterial Enhancement Reagent (Invitrogen), pre-heated to 95°C. Cells were lysed by incubation at 95°C for 5 min and cooled to RT. TRIzol™ reagent (800 μl) was added to lysates and vortexed for 10 s. Following centrifugation (14 000 × *g*, 5 min, 4°C), the liquid phase was transferred to tubes containing 200 μl chloroform, mixed by vortexing, and centrifuged (17 200 × *g*, 5 min, 4°C). The aqueous layer was re-extracted with chloroform, and RNA precipitated by addition of 1 volume ice-cold isopropanol and 0.1 volume of 7.5 M ammonium acetate. RNA was pelleted by centrifugation (21 000 × *g*, 30 min, 4°C) and washed with 100% and then 70% ethanol. RNA was resuspended in 20 μl of nuclease-free water and incubated at 37°C with 2.7 U RNase-free DNase I. Following heat inactivation of DNase I, RNA concentrations were measured using the Qubit™ kit (Thermo Fisher Scientific).

For generating complementary DNA (cDNA), 350 ng of RNA was reverse transcribed using HiScript IV qRT Supermix (Vazyme) according to the manufacturer’s instructions. Concentrations of *hsdM* mRNA and 16S rRNA (housekeeping control) were quantified by digital droplet PCR (ddPCR). Briefly, ddPCRs were prepared containing 1× QX200™ ddPCR™ EvaGreen Supermix (Bio-Rad) and 10 mM of each primer (16S rRNA, primers YZ435/YZ436; *hsdM*, primers YZ437/YZ438). Serial dilutions of cDNA were subsequently added to reactions. Droplets were generated in DG8™ ddPCR cartridges containing 70 μl QX200™ Droplet Generation Oil (Bio-Rad) per reaction. Generated droplets were transferred into 96-well plates and genes amplified in a thermocycler (95°C, 5 min; 39×: 95°C, 30 s, 60°C, 1 min; 4°C, 4 min; 90°C, 5 min; 4°C hold). Positive and negative droplet event counts were measured in a QX200™ ddPCR droplet reader, and absolute concentrations of *hsdM* positive normalized to positive droplet counts for the 16S rRNA and expressed as a fold change relative to the *t* = 0 time point.

### Gene neighbourhood analysis

Gene neighbourhood analysis was performed using webFlaGs (https://server.atkinson-lab.com/webflags) [45] with the EPEC RptR amino acid sequence as input query. Default settings were applied with the following exceptions: number of flanking genes, 10; and maximum number of BLAST hits, 200. webFLaGs was run on two independent occasions with the same search parameters on the following dates: Run 1 (12 December 2024) and Run 2 (2 May 2025). Clusters of *rptR*-associated neighbouring genes for both runs are listed in Supplementary Tables S7 and S8, respectively. Output data for both runs were collated to give a total of 117 unique RptR homologues. Of these, 87 homologues gave full-length RptR sequences, extracted from corresponding NCBI accession numbers from webFlaGs, which were then aligned using NCBI COBALT. Promoter sequences, within 100 bp upstream of *rptR* start codons, were obtained for a total of 80 RptR homologues and were examined for the presence/absence of predicted RptR binding sites, defined as any of TAGCTA, TAGATA, TATCTA, or TATATA. Sequence logos were generated using the WebLogo tool [46].

### AlphaFold structural predictions

Structural predictions of dimeric RptR with or without 9-bp dsDNA (TTTAGCTAA) were obtained using the AlphaFold 3 web server (https://alphafoldserver.com/) [47]. Predicted protein structures and interfaces were considered to be confident if pTM and ipTM values were above 0.8, respectively. Structural models were visualized using PyMOL (The PyMOL Molecular Graphics System, Version 3.10, Schrödinger, LLC).

### Statistical analysis

All statistical analyses were performed in GraphPad Prism. Data points are given as mean ± SD (standard deviation) with statistically significant differences (*P <* .05) determined using Student’s *t*-test (between two groups), one-way analysis of variance (ANOVA) with post-hoc Tukey’s test or Dunnett’s test (between more than two groups), or two-way ANOVA with post-hoc Šidák’s test (between more than two categories).

## Results

### A seven-ORF defence island in EPEC confers anti-phage immunity

**Figure 1.**
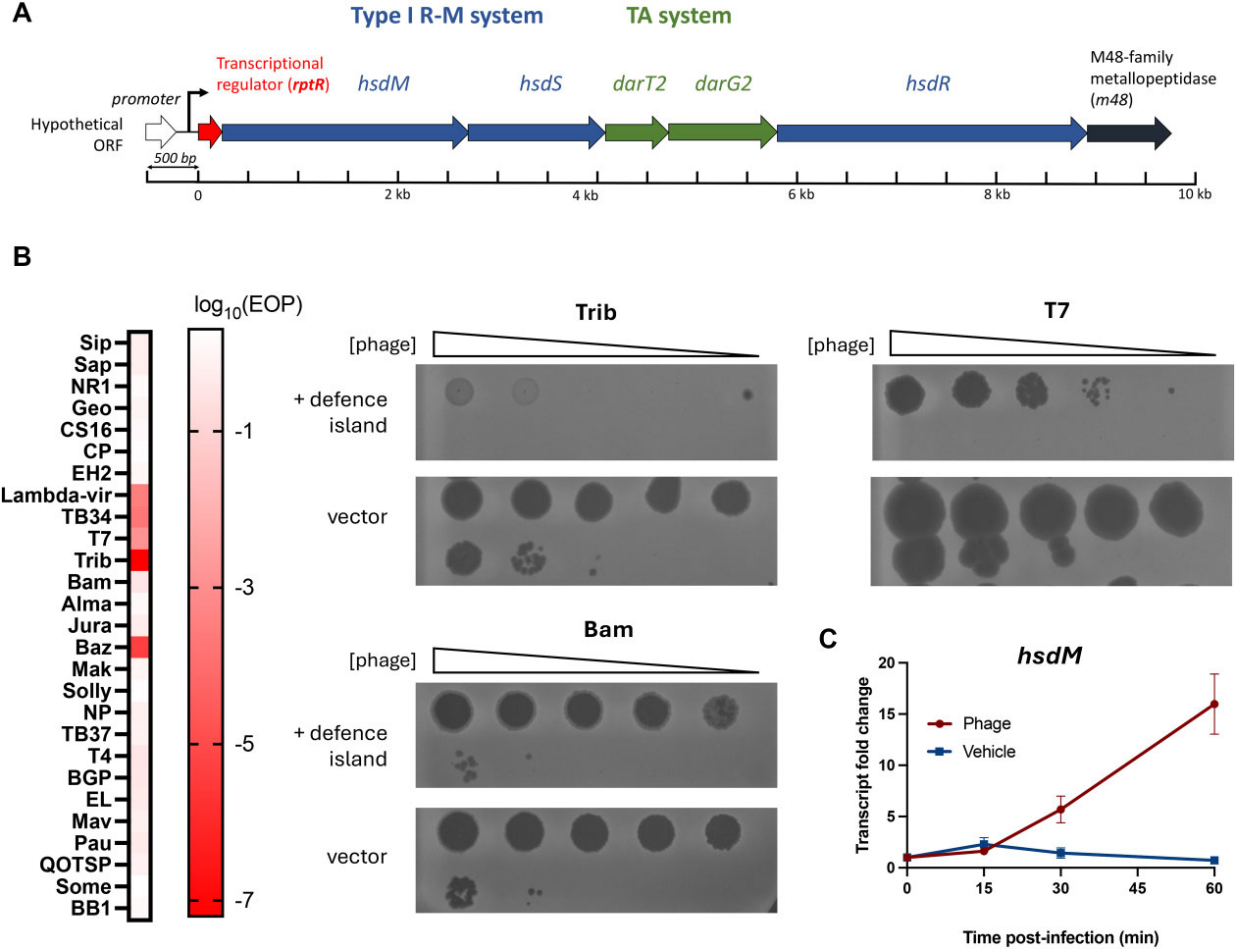
The RptR-RMS-DarTG-M48 (RRDM) defence island from EPEC confers protection against phage. (**A**) Schematic of the defence island composed of seven ORFs encoding a Type I restriction–modification (R-M) system (HsdM: WP_000985463.1, HsdS: WP_000228010.1, HsdR: WP_000108733.1), a TA system (DarTG2) (DarT: WP_000312835.1, DarG: WP_000634203.1), a predicted M48-family metallopeptidase (WP_001122107.1), and a putative transcriptional regulator (WP_000343040.1). (**B**) Heatmap of phage efficiency of plaquing (log_10_(EOP)) on *E. coli* DH5α carrying pBR322-RptR-RMS-DarTG-M48 (+ defence island) or empty vector. Examples are shown of phage plaque assays in which the defence island confers protection (Trib, Barry), partial protection (T7), or no protection (Bam). Ten-fold serial dilutions of phage were spotted on bacterial lawns either expressing the full defence island or an empty vector. (**C**) *hsdM* relative transcript levels during infection by Trib phage. Bacterial cells were collected at 15, 30, and 60 min post-infection, RNA extracted, and transcript levels for *hsdM* quantified by digital droplet PCR. Values represent mean fold changes in *hsdM* transcript levels, after normalization to 16S rRNA levels, ± SD of *n* = 3 biological replicates.

In the EPEC strain E2348/69 [48], DarTG2 (WP_000312835.1, WP_000634203.1) is embedded within a Type I RMS (HsdM: WP_000985463.1, HsdS: WP_000228010.1, HsdR: WP_000108733.1), between genes encoding the specificity (HsdS) and endonuclease (HsdR) subunits (Fig. 1A). Further examination of the genomic context of the EPEC RMS–DarTG systems shows they are flanked upstream by an ORF encoding a putative transcriptional regulator (WP_000343040.1, here designated *rptR*) and downstream by a gene encoding a predicted M48-family Zn^2+^ metallopeptidase (WP_001122107.1) (Fig. 1A). The seven-ORF island is flanked by insertion sequences (IS) of the IS*100*, IS*3*, and IS*66* families, suggesting that it was acquired by horizontal gene transfer. As anti-phage defence systems are commonly co-localized in genetic islands [19], we hypothesized that this EPEC island could have a role in protection against phage infection.

To investigate whether the EPEC island has anti-phage activity, we placed all seven genes of the EPEC island under the control of their native promoter(s) in a pBR322 vector (pBR322-RRDM). We tested *E. coli* DH5α containing the vector with or without the island for sensitivity to 24 phages from the Durham collection [34] as well as three well-characterized coliphages (i.e. Lambda-*vir*, T4, and T7) (Fig. 1B). The *E. coli* DH5α host strain background was used due to its known susceptibility to phages of the Durham collection, originally isolated on the same strain [34]. The relative efficiency of plating (EOP) was expressed as the ratio of phage plaques on the host strain carrying the defence island versus the host strain carrying the empty vector; EOP values below 1 indicate anti-phage protection. Five phages gave EOP of <1 in the presence of the island: Trib, Barry (Baz), T7, TB34, and Lambda-*vir* (Fig. 1B). Protection was observed against T7 phage, with a 1000-fold lower EOP on hosts with the island than in those carrying the empty vector (Fig. 1B). In contrast, the island conferred protection of over seven orders of magnitude against phages Trib and Baz (Fig. 1B), which share 99.1% nucleotide sequence identity [34]. Protection against Lambda-*vir* and TB34 was between these values, at around four orders of magnitude (Fig. 1B). Growth of the host strain in the presence of the EPEC island and Trib phage with MOI = 1 was indistinguishable from cultures in the absence of phage, consistent with the island conferring immunity against Trib infection (Supplementary Fig. S1). Furthermore, we assessed mRNA levels of *hsdM*, the first defence component in the island, in response to phage infection and found it was upregulated by over 15-fold within 1 h following infection by Trib phage at a high MOI; transcript levels of *hsdM* were virtually unchanged in uninfected bacteria (Fig. 1C). Therefore, we named this EPEC island RRDM (RptR-RMS-DarTG-M48), as it is a functional anti-phage defence island.

### An active promoter is present upstream of RRDM

**Figure 2.**
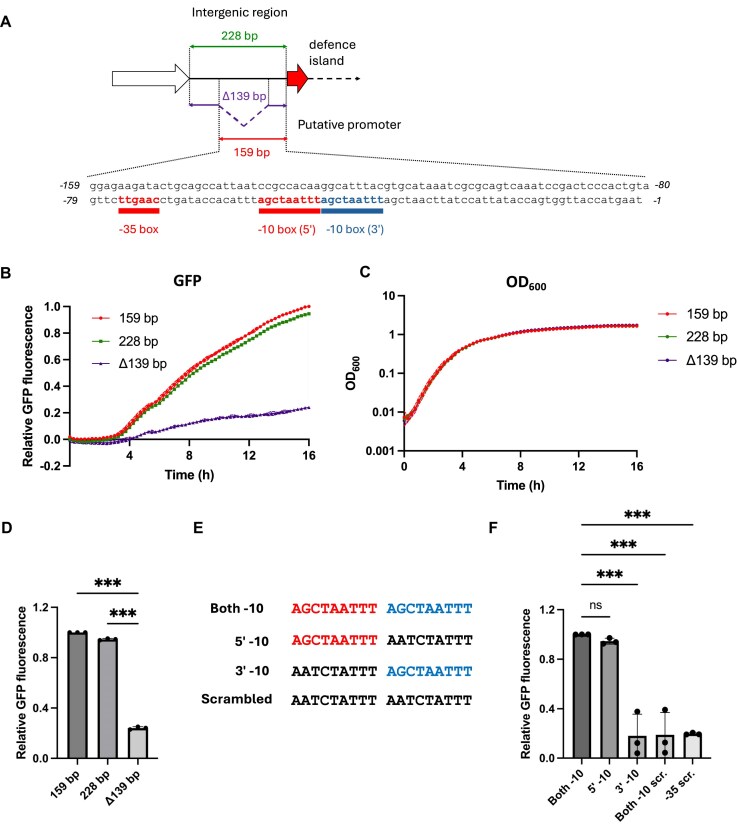
An active promoter is found within 159 bp upstream of the defence island. (**A**) Schematic of the putative *rptR* promoter and sequence 159 bp upstream of the first start codon of the defence island. Sequence regions tested for promoter activity (159 bp, 228 bp, Δ139 bp) are highlighted. Predicted −35 (red) and two −10 boxes (red and blue) are highlighted. GFP transcriptional reporter assay in *E. coli* MG1655. GFP, on pBR322, was expressed under the control of the indicated sequences and GFP absorbance (495 nm) (**B**) or OD_600_ (**C**) measurements taken every 15 min for 16 h. Values represent mean with SD (shaded) of *n* = 3 biological replicates. (**D**) Bar chart of relative GFP fluorescence at 16 h post-inoculation. Values represent mean ± SD of *n* = 3 biological replicates. ***P < .001 (one-way ANOVA with Dunnett’s test for multiple comparisons). (**E**) Sequences of adjacent −10 boxes in promoters that contain both, either, or neither 5′ (red) nor 3′ (blue) −10 boxes. (**F**) Bar chart of relative GFP fluorescence intensity at 16 h post-inoculation for promoters containing both, either, or neither −10 boxes nor scrambled −35 box. Values represent mean ± SD of *n* = 3 biological replicates. ***P < .001; ns, not significant (one-way ANOVA with Dunnett’s test for multiple comparisons).

Although the mechanisms of anti-phage protection conferred by the Type I RMS and DarTG systems are understood, it is unknown how they are regulated as part of an integrated defence island, such as in RRDM. As expression of *hsdM* is upregulated upon phage infection (Fig. 1C), we first sought to identify potential promoter sequences upstream of RRDM. Using an automated bacterial promoter sequence predictor [49], we identified one −35 (TTGAAC) and two identical −10 boxes (AGCTAATTT) at 72, 51, and 42 bp upstream of the predicted *rptR* start codon, respectively (Fig. 2A). To determine whether these sequences act as a promoter, we employed a GFP reporter assay. GFP was cloned in pBR322 downstream of either the entire 228-bp intergenic sequence upstream of the island, only 159 bp of sequence upstream of the island, or a sequence derived from the intergenic sequence lacking a 139-bp region containing the predicted −35 and −10 boxes (Δ139-bp) (Fig. 2A). Levels of fluorescence of *E. coli* MG1655 carrying the GFP reporter fusions were measured over 16 h. Strains containing GFP constructs with either the 228-bp or 159-bp sequence gave robust GFP expression over 16 h (Fig. 2B–D). In contrast, GFP expression from the Δ139 bp sequence was ∼5-fold lower than from reporter strains carrying the predicted promoter motifs (at 16 h: versus 159 bp, *P*<.001; versus 228 bp, *P <*.001) despite no change in cell growth compared with strains carrying 228- or 159-bp sequences (Fig. 2C), indicating that the observed lower GFP expression is due to loss of promoter activity. Thus, the 139-bp sequence encompassing both −10 and −35 boxes likely contains a functional promoter upstream of RRDM.

As the potential RRDM promoter contains two identical −10 boxes (at 51 and 42 bp upstream of the predicted *rptR* start codon), we next determined which element is required for promoter activity. We used the pBR322-GFP reporter construct carrying the 159-bp sequence and scrambled either the 5′ (51 bp) or 3′ (42 bp) −10 box, both −10 boxes, or the −35 box (Fig. 2E and F). Promoters containing only the 5′ −10 box were sufficient to drive GFP expression at similar levels to promoters containing both −10 boxes (Fig. 2F). Promoters containing only the 3′ −10 box gave GFP expression levels similar to the Δ139 bp sequence that lacks both −10 boxes (Fig. 2F). Scrambling of the −35 box, with both −10 boxes intact, also attenuates GFP expression (Fig. 2F). Therefore, the activity of promoter sequences upstream of RRDM is dependent on the 5′ −10 box located 51 bp upstream of *rptR* (Fig. 2A), with the motif separated by 14 bp from the functional −35 box.

### RptR represses its native promoter

Attempts to clone *hsdMSR* or *darTG2* directly downstream of the RRDM promoter in pBR322 proved unsuccessful; all clones contained predicted inactivating mutations or truncations. We hypothesized that this resulted from toxicity of uncontrolled expression of the systems, which suggests a need for their regulation. The first ORF of the RRDM island is predicted to encode a small transcriptional regulator, based on automated annotation, which does not belong to any known class of regulator based on sequence similarity. Therefore, we decided to characterize the function of this protein we termed RptR.

Initially, to understand the multimerization state of RptR in solution, we purified wild-type (WT) RptR with an N-terminal hexahistidine (His_6_) tag and 3C protease cleavage site. WT RptR gave a single species eluting at 12.6 ml on a Superdex 75 Increase column (Supplementary Fig. S2A). This peak contained a major band of ∼11 kDa by SDS–PAGE, matching the expected MW for tagged monomeric RptR (Supplementary Fig. S2B). Following analytical SEC using a mixture of five calibration standard proteins, the MW of purified RptR in solution was interpolated to be ∼25.3 kDa (*R*^2^ = 0.99; theoretical weight of His_6_-3C-RptR: 22.8 kDa), consistent with RptR being predominantly dimeric in solution (Supplementary Fig. S2C and D).

**Figure 3.**
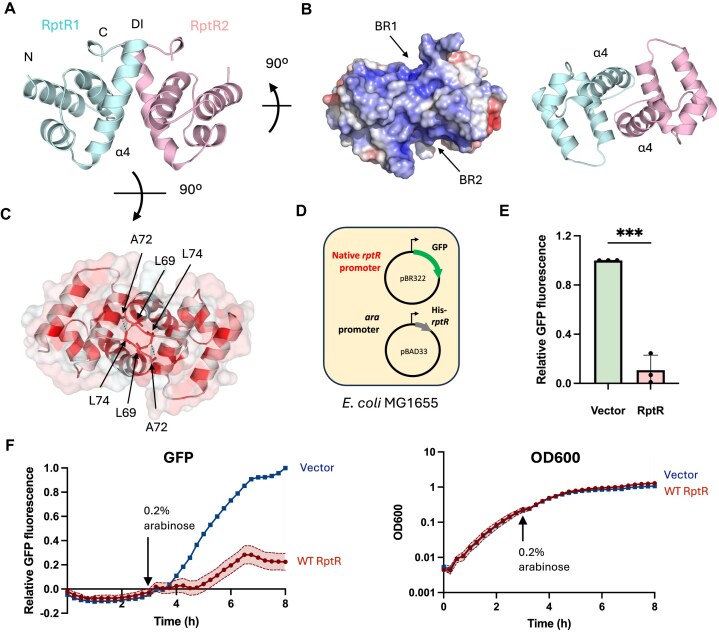
RptR is a small transcriptional repressor of its native promoter. (**A**) Crystal structure of RptR dimer with monomers highlighted. Positions of N- and C-terminal residues are indicated alongside helix α4 and the dimerization interface (DI). (**B**) Surface electrostatics of RptR structure from panel (A) showing two basic regions (BR1 and BR2) on the underside of the dimer (left) and corresponding cartoon representation showing positions of α4 helices (right). (**C**) Top view showing dimerization interface of RptR coloured according to the Eisenberg hydrophobicity scale [41]. Selected residues Ala72 (A72), Leu69 (L69), and Leu74 (L74) from each monomer are highlighted. (**D**) Schematic of a dual-plasmid GFP transcriptional reporter system in *E. coli* MG1655 with arabinose-inducible expression of RptR *in trans* on pBAD33. (**E**) GFP transcriptional reporter assay with expression of RptR *in trans*. Values represent mean GFP fluorescence intensities relative to vector control ± SD of *n* = 3 biological replicates at 16 h post-inoculation. ***P < .001 (Student’s *t*-test). (**F**) GFP transcriptional reporter time course assay after induction of RptR at 3 h post-inoculation. Values represent mean GFP fluorescence intensities relative to vector control (left) or cell density, measured as absorbance at 600 nm (OD_600_), (right) with SD (shaded) of *n* = 3 biological replicates.

To obtain further insights into the function of RptR, we solved its crystal structure to 1.3 Å (PDB ID: 9R2Z) using a model of dimeric RptR generated by AlphaFold 3 for molecular replacement [47] (Fig. 3A–C and Supplementary Fig. S3). Further data collection and refinement statistics are listed in Table 1. Consistent with analytical SEC, RptR formed a homodimer of two helical monomers each comprising four alpha helices (α1–α4) (Fig. 3A). Each monomer presents an extended α4 helix splayed out from the dimerization interface at the C-terminus (Fig. 3A); the intervening surface consisting of the two α4 helices forms two basic (positive electrostatic) regions that likely present the DNA binding site of RptR (Fig. 3B). Although structural homology searches using Foldseek [50] and DALI [51] with the RptR structure did not identify any close structural homologues in the PDB, the arrangement of helices α2–α4 is reminiscent of the tri-helical motif of HTH transcriptional regulators [52] that are also dimeric. This is reflected in the inclusion of a TetR-like HTH transcriptional regulator from *Streptomyces* (PDB ID: 2NP3) returned in the top 5 structural homologues of RptR from DALI, despite sharing only 7% sequence identity with RptR (Supplementary Table S4).

**Table 1.** Crystallographic data collection and refinement statistics

Structure	RptR apo
PDB ID	9R2Z
Data collection	
Space group	*P*2_1_
Unit cell parameters	
* a* (Å)	30.6
* b* (Å)	41.5
* c* (Å)	63.3
* α* (*°*)	90
* β* (°)	90.1
* γ* (°)	90
Wavelength (Å)	0.9537
Resolution range	34.72–1.30 (1.34–1.30)
Completeness (%)	100.00 (99.80)
Redundancy	6.7 (2.0)
CC half	0.999 (0.870)
* R*_merge_ (%)	1.9 (34.7)
* I/σ*(*I*)	12.1 (1.9)
Refinement	
Resolution (Å)	34.72–1.30 (1.34–1.30)
No. of unique reflections	39029 (2966)
* R*_work_/*R*_free_	0.171/0.193
No. of non-H atoms	
Macromolecules	1256
Solvent	194
RMS deviations	
Bond length (Å)	0.005
Bond angle (°)	0.8
Ramachandran outliers (%)	0
Rotamer outliers (%)	0
Clashscore	2.39

Values in parentheses relate to the high-resolution shell.

In contrast to RptR, the dimerization interfaces of HTH regulators are typically distal to the DNA-binding domain [53–55]. In RptR, dimerization is mediated through hydrophobic interactions between residues in helix α4 and the unstructured C-terminus of the protein (i.e. residues L74, L69, R68, E25, and L65; Fig. 3C, Supplementary Fig. S3A, and Supplementary Table S5). Leu74 of one RptR monomer interacts with Leu69 and Ala72 from the other monomer, and vice versa (Fig. 3C). Dimerization is also further stabilized by residue contacts that are more distal from the C-terminus including a key electrostatic interaction between Glu25 and Arg68 and a hydrophobic interaction between Val24 and Leu65 (Supplementary Fig. S3A). To disrupt this dimerization interface, we attempted to purify RptR with a substitution of Leu74 to arginine (L74R), preventing hydrophobic interactions of Leu74 with Leu69 and Ala72 by replacing it with a charged, sterically bulky residue. Purified RptR L74R could not be detected following SEC (Supplementary Fig. S2A), potentially due to protein destabilization upon disruption of dimerization. This aligns with our structural data demonstrating the dimeric nature of RptR mediated through a combination of hydrophobic and electrostatic interactions.

To determine whether RptR regulates its own promoter, we generated a dual-plasmid GFP reporter system (Fig. 3D); pBR322 encoding GFP downstream of the 159-bp RRDM promoter was introduced into *E. coli* MG1655 harbouring pBAD33 with His_6_-RptR under the control of an arabinose-inducible promoter. Cells expressing WT RptR *in trans* gave ∼10-fold lower GFP expression than control cells carrying the empty pBAD33 after 16-h growth (Fig. 3E). Furthermore, addition of the inducer at 3 h post-inoculation resulted in plateauing of the GFP signal in the strain expressing RptR with no corresponding change in cell growth, in contrast with the strain containing the empty vector that gave increasing fluorescence signal over the subsequent 5 h (Fig. 3F). We therefore conclude that RptR represses its native promoter.

### RptR binds to direct repeats in the RRDM promoter

**Figure 4.**
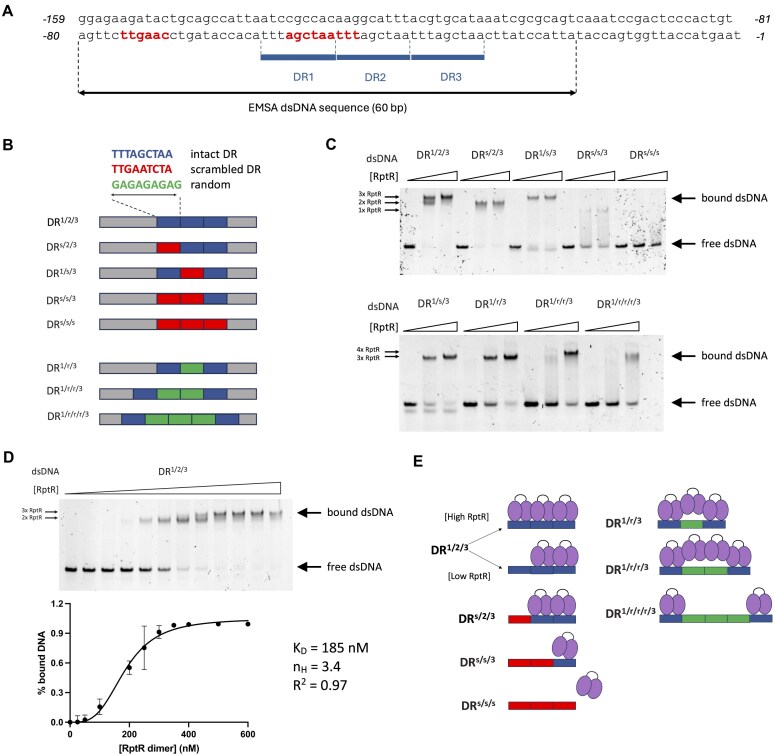
RptR binds in a modular fashion to 9-bp imperfect direct repeats in its native promoter. (**A**) Sequence of defence island promoter with three direct repeats (DRs): TTTAGCTAA (bars). Bases corresponding to the −35 and −10 boxes are highlighted. The WT dsDNA sequence (60 bp) used in subsequent EMSAs is also indicated. (**B**) Schematic of 60-bp dsDNA sequences used in EMSAs. Coloured bars represent DRs that are intact (blue), scrambled (red), or replaced by a random 9-bp sequence (green). (**C**) EMSA of WT RptR protein, to final concentrations of 0, 0.6, and 1.2 μM, incubated with 60-bp dsDNA (0.05 μM) containing combinations of intact and scrambled DRs (upper) or intact DRs and random 9-bp sequences (lower). Images are representative of *n* = 2 independent experiments. (**D**) EMSA of WT RptR protein, to final monomer concentrations of 0, 0.025, 0.05, 0.1, 0.2, 0.4, 0.5, 0.6, 0.7, 0.8, 1.0, and 1.2 μM, incubated with 60-bp dsDNA (0.05 μM) containing three intact DRs (upper). Images are representative of *n* = 5 independent experiments. Binding curve of RptR dimers to DR^1/2/3^ obtained from quantification of EMSA free DNA bands. Values represent mean ± SD of *n* = 5 independent experiments. Binding parameters are indicated: *K*_D_, dissociation constant; *n*_H_, Hill coefficient; *R*^2^,coefficient of determination. (**E**) Proposed binding model of RptR to dsDNA sequences containing combinations of intact, scrambled, or random 9-bp DR sequences used in EMSAs.

As RptR expressed *in trans* represses the activity of the RRDM promoter, we hypothesized that it binds directly to the promoter and blocks recruitment of the transcriptional machinery. Because RptR is likely to function as a homodimer, we expected the RptR binding site(s) to be palindromic with each monomer of RptR recognizing the same sequence motif. Therefore, we scanned the 159-bp sequence upstream of *rptR* for palindromes. We identified a series of three 9-bp DRs (DR1 to DR3), each containing the 6-bp palindrome TAGCTA, located between 58 and 31 bp upstream of the *rptR* start codon (Fig. 4A). Of note, DR1 and DR2 overlap with the 5′ −10 box of the RRDM promoter (Fig. 4A).

To determine whether RptR binds specifically to these DRs, we performed EMSAs using purified RptR and DNA fragments corresponding to regions of the *rptR* promotor. We incubated increasing concentrations (0, 0.6, and 1.2 μM) of RptR with 60-bp dsDNA sequences containing either three intact DRs (DR^1/2/3^) or three scrambled DRs (DR^s/s/s^; Fig. 4B). While no RptR binding was detected to DR^s/s/s^, two stepwise band shifts of DR^1/2/3^ were observed with increasing RptR concentration (Fig. 4C). These data indicate that RptR binds directly to its promoter with either full or partial occupancy of the three DRs present.

To test whether RptR can bind to promoter sequences containing fewer than three DRs, we performed EMSAs with dsDNA in which DR1 was scrambled (generating two adjacent DRs, DR^s/2/3^), DR2 was scrambled (generating separated DRs, DR^1/s/3^), or both DR1 and DR2 were scrambled (DR^s/s/3^) (Fig. 4B). Band shifts were observed with both DR^s/2/3^ and DR^1/s/3^ consistent with RptR binding to dsDNA containing two DRs (Fig. 4C). Of note, when intact DRs were positioned next to each other (in DR^s/2/3^), the resulting single band shift matched the lower shift seen with DR^1/2/3^. In contrast, only the higher band was observed with DR^1/s/3^, which contains two intact DRs separated by a scrambled sequence (Fig. 4C). dsDNA containing a single intact DR (DR^s/s/3^) displayed an even lower band shift with a substantially weaker intensity than that seen with DR^s/2/3^ indicating RptR binds poorly to a single DR (Fig. 4C).

The presence of only the higher band shift when the middle DR is scrambled (DR^1/s/3^) suggests that there is binding of a third RptR homodimer when RptR homodimers are bound to intact DR1 and DR3. In this scenario, the higher band would be observed irrespective of the intervening sequence, as the third RptR homodimer would be mainly stabilized by protein–protein, and not protein–DNA, interactions. Consistent with this, we found that replacement of the scrambled DR2 in DR^1/s/3^ with a random 9-bp sequence (GAGAGAGAG, DR^1/r/3^) produced the same band shift as DR^1/s/3^ (Fig. 4C). Addition of a further intervening random 9-bp sequence (DR^1/r/r/3^) produced a single, higher shift but only at the highest RptR concentration (1.2 μM). However, addition of three intervening random 9-bp sequences (DR^1/r/r/r/3^) resulted in a lower shift with faint bands (Fig. 4C). This may result from the increased distance between the two intact DRs preventing stabilising interactions between bound RptR homodimers.

To determine whether the observed binding of multiple RptR dimers to the three adjacent DRs in the WT promoter (DR^1/2/3^) is cooperative, we quantified free DNA bands from EMSAs where the same concentration of dsDNA was incubated with more intermediate concentrations of RptR (Fig. 4D). From the obtained binding curve (*R*^2^ = 0.97), the mean Hill coefficient (*n*_H_ = 3.4) was consistent with positive cooperativity in RptR binding to three distinct sites (Fig. 4D). The equilibrium dissociation constant (*K*_D_) for dimeric RptR binding was estimated at 185 nM (95% CI: 171–198 nM) indicative of relatively low DNA binding affinity comparable to the cI HTH repressor (80 nM) [56], in contrast to the high-affinity binding profiles of HTH regulators with substantially longer DNA binding sites, e.g. the lac repressor (2 pM) [57]. Collectively, these data suggest a model in which RptR homodimers bind in a modular and cooperative fashion to an array of at least two DRs to effect transcriptional repression (Fig. 4E).

**Figure 5.**
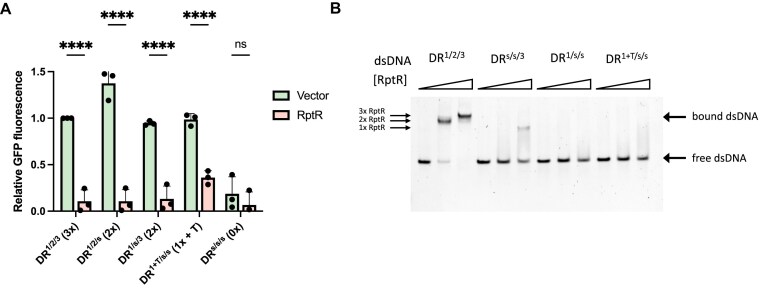
Full RptR transcriptional repression requires two intact direct repeats. (**A**) GFP transcriptional reporter assay. GFP was expressed downstream of promoters containing the indicated combinations of intact and scrambled DRs in the presence or absence of RptR expressed *in trans*. Values represent mean ± SD of *n* = 3 biological replicates. ****P < .0001; ***P < .001; ns, not significant (two-way ANOVA with Šidák’s multiple comparisons test). (**B**) EMSA of WT RptR protein, to final concentrations of 0, 0.6, and 1.2 μM, incubated with 60-bp dsDNA (0.05 μM) containing either three DRs (DR^1/2/3^), one intact DR at the positions indicated, or one intact DR with a thymidine at position T3 of DR2 as found in the DR^1+T/s/s^ promoter in panel (A). Images are representative of *n* = 2 independent experiments.

As sequences containing two DRs bind RptR as effectively as three DRs, we hypothesized that promoters with this sequence would be repressed by RptR *in vivo*, similar to the WT promoter. To test this, we scrambled the sequence of either DR3 (DR^1/2/s^) or DR2 (DR^1/s/3^), leaving only two intact DRs, in the promoter of the pBR322-GFP reporter plasmid and examined the activities of the resulting promoters using the dual-plasmid reporter assay. As expected, both promoters carrying two DRs were repressed upon induction of RptR to a similar extent as the WT promoter (Fig. 5A). In contrast, the promoter containing a single DR (DR^1+T/s/s^), and an adenine to thymine substitution at the T3 position in the scrambled DR2 to maintain a functional −10 box, gave reduced RptR-dependent gene repression compared with promoters containing two or three DRs (Fig. 5A). This A-T substitution at position T3 did not restore RptR binding in EMSAs with binding of RptR comparable to dsDNA containing only a single DR (DR^1/s/s^) (Fig. 5B). However, as GFP expression was still significantly reduced in the DR^1+T/s/s^ promoter compared with the empty vector strain, some RptR-dependent promoter repression was still present (Fig. 4E). Thus, despite the presence of three DRs in the RRDM promoter, two DRs are sufficient for RptR-mediated repression *in vivo* with only partial repression observed with one DR.

### RptR binds a TAGCTA palindromic sequence

**Figure 6.**
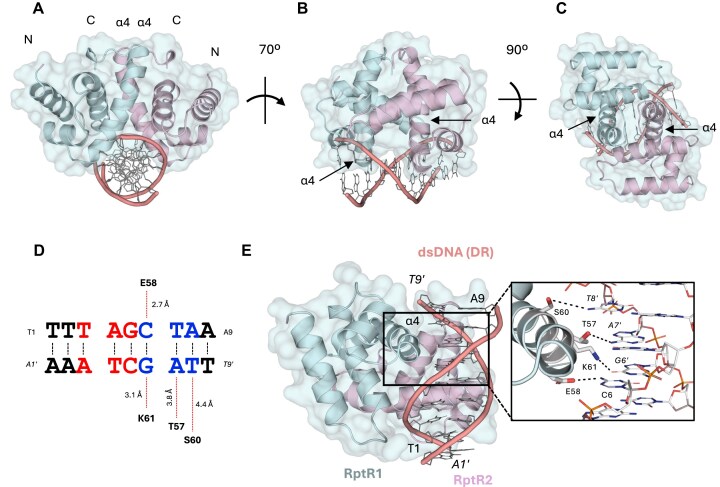
Structural models of dimeric RptR bound to a 6-bp palindrome. (A–C) AlphaFold3 models of dimeric RptR (RptR1/2) bound to 9-bp dsDNA (TTAGCTAA) displayed as a front view analogous to Fig. 3A (**A**), a side-view with the dsDNA duplex running horizontally (**B**), or a top view (**C**). (**D**) Schematic showing the predicted 6-bp palindrome, within each 9-bp DR, recognized by RptR DNA-binding residues. The inter-atomic distances between each residue–base interaction pair (dashed line) is shown. (**E**) Underside view of RptR dimer bound to dsDNA in an analogous orientation to Fig. 3B. A predicted interaction interface is shown between residues of the RptR DNA recognition helix and dsDNA bases (inset box). Residue–base interactions are represented by dashed lines.

As RptR binding to dsDNA *in vitro* depends on the 9-bp DR sequence TTTAGCTAA, we sought to define the minimal RptR recognition motif. As the crystal structure of RptR bound to DNA could not be obtained and given the very high similarity between the crystal structure and the predicted model of RptR in its apo state (RMSD = 0.50 Å, Supplementary Fig. S3B), we used AlphaFold 3 to model homodimeric RptR bound to the 9-bp DR sequence. In this model, the α4 helices in a RptR dimer, which form two basic clefts in the apo structure (Fig. 3B), form the main DNA-binding interface and insert into either end of a single major groove of DNA (Fig. 6A–C). This unusual arrangement is enabled by the splayed conformation of the two α4 helices in a RptR dimer that are predicted to straddle the DNA duplex and together form contacts, via the N-terminal residues of helix α4, with a short TAGCTA 6-bp palindrome, numbered from T3 to A8 within the wider 9-bp DR sequence (Fig. 6D and E). Each α4 helix was modelled to interact with a 3-bp half-site within the 6-bp palindrome (Fig. 6D and E).

**Figure 7.**
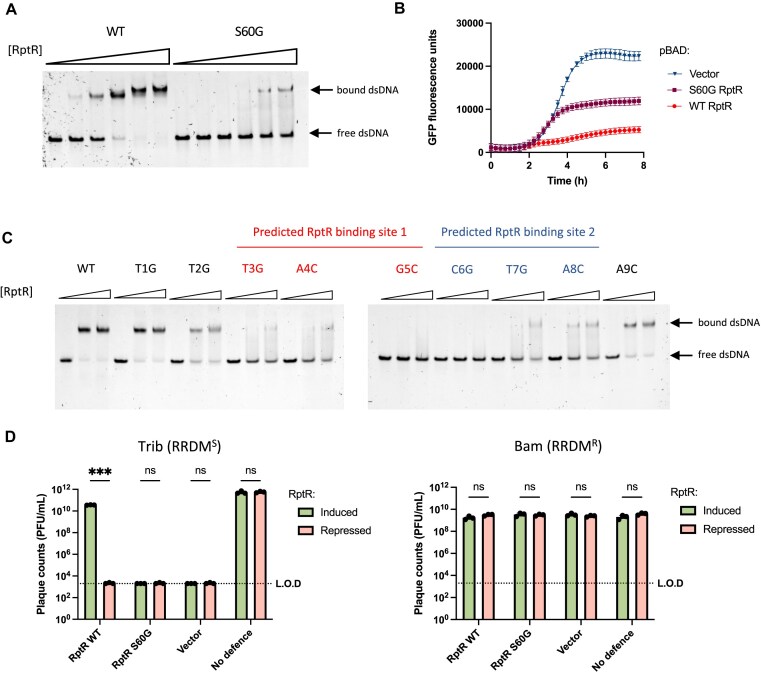
RptR recognizes a central 6-bp palindrome, TAGCTA. (**A**) EMSA of WT or DNA binding-deficient (S60G) RptR protein (0, 0.15, 0.3, 0.6, 0.9, and 1.2 μM) incubated with 60-bp dsDNA containing all three DRs (WT) (Fig. 4B). Image representative of *n* = 2 independent experiments. (**B**) GFP transcriptional reporter assay. GFP was expressed downstream of the native RRDM promoter with *in* *trans* expression of WT or S60G RptR, or an empty pBAD33 vector. Values represent mean ± SD of *n* = 3 biological replicates. (**C**) EMSAs of WT RptR protein (0, 0.6, and 1.2 μM) incubated with 60-bp dsDNA (0.05 μM) containing two adjacent IRs (IR^1/2/s^). Both IRs in each dsDNA sequence, except the WT control, contain base substitutions at the indicated positions. Base positions within the 6-bp palindrome predicted to be recognized by each RptR monomer are highlighted (Predicted RptR binding site 1/2). Images are representative of *n* = 2 independent experiments. (**D**) Quantification of plaque assays of either an RRDM-sensitive (RRDM^S^) phage, Trib (left), or an RRDM-resistant (RRDM^R^) phage, Bam (right), on *E. coli* DH5α carrying pBR322-RRDM and with either arabinose induction or glucose repression of RptR (WT or S60G) expressed from pBAD33 vectors. Vector denotes the strains carrying empty pBAD33 and an inactive RRDM control strain (No defence) was included carrying pBR322-RRDM with an F267A mutation in HsdM and pBAD33 expressing WT RptR. Bars represent mean ± SD of *n* = 3 biological replicates. ***P < .001; ns, not significant (unpaired Student’s *t*-test with Bonferroni–Dunn correction for multiple comparisons). LOD, limit of detection.

At this interface, a predicted network of hydrogen bonds between RptR and DNA was identified from residues Thr57 to Lys61, inclusive (Fig. 6D and E). To test this, we purified RptR containing a Ser60 to glycine mutation (S60G) with this residue position selected due to it having the longest predicted hydrogen bond length to a DNA base in the AlphaFold 3 model, and therefore the smallest expected effect size upon mutagenesis (Fig. 6D). RptR S60G showed reduced binding to dsDNA containing all three DRs (DR^1/2/3^) compared to WT RptR, with unbound dsDNA still detected at the highest concentration of protein used (Fig. 7A). The reduced DNA binding of S60G RptR correlates with its reduced ability to repress the promoter *in vivo* in the dual-plasmid GFP reporter assay (Fig. 7B).

To test our model that RptR recognizes the central 6-bp palindrome TAGCTA, we introduced single point mutations at each position in the 9-bp DR (i.e. T1 to A9 inclusive) in all three DRs within a 60-bp dsDNA fragment and performed EMSAs to assess protein–DNA interactions; each base in the 9-bp DR sequence was systematically replaced according to a mutational key (i.e. thymidine with guanine, adenine with cytosine, and vice versa; Supplementary Fig. S4A). Unexpectedly, *in vitro* binding of His_6_-3C-RptR was unaffected by the introduction of any such point mutation in DR^1/2/3^ (Supplementary Fig. S4B). However, further mutation of cytosine C6 to guanine (C6G), instead of C6A, did abolish RptR binding (Supplementary Fig. S4C), suggesting that this base position is essential for RptR binding albeit with redundancy between cytosine and adenine.

To investigate the structural basis for this redundancy, we used AlphaFold 3 to model an RptR homodimer bound to a 9-bp DR containing G6 or A6 (instead of C6). In these models, Glu58 (E58) of RptR was predicted to form a hydrogen bond with the amino group of A6 that resembles the E58–C6 interaction (Supplementary Fig. S4D). Meanwhile, with G6, E58 faces the electronegative carbonyl group at carbon-6 with which it is unlikely to interact (Supplementary Fig. S4D). This suggests that C6 and A6 should be similarly tolerated in the RptR binding site consistent with the experimental RptR–DNA binding data (Supplementary Fig. S4D). As the 6-bp predicted binding site is a perfect palindrome, such redundancy would also apply to the G5/C5′ base pair binding, at an analogous position as C6/G6′, to the other RptR protomer in the homodimer (Fig. 6D).

As RptR binding to DR^1/2/3^ was largely unaffected by point mutations, we attempted to identify base substitutions influencing RptR binding by removing one DR. We repeated the mutational analysis and EMSAs with dsDNA containing two adjacent DRs (DR^1/2/s^), which gives a single band shift with RptR. With these constructs, WT RptR binding was substantially reduced upon mutation of any base in the central 6-bp palindrome; smaller effects on RptR binding were observed with mutations at positions T1, T2, and A9 (Fig. 7C). Thus, we propose the RptR recognition sequence is a 6-bp TAGCTA palindrome with some flexibility at positions G5 and C6.

We next determined whether constitutive transcriptional repression mediated by RptR is sufficient to impair anti-phage protection *in vivo*. To test this, we generated a dual-plasmid strain of *E. coli* DH5α carrying pBR322-RRDM and pBAD33-His_6_-RptR (WT/S60G) enabling arabinose-inducible overexpression of RptR. Upon glucose repression of WT RptR expression, RRDM provided robust protection against Trib phage (Fig. 7D). However, upon induction of WT RptR, anti-phage protection was abrogated with *E. coli* strains re-sensitized to Trib phage (Fig. 7D). This re-sensitization was dependent on RptR–DNA binding as the S60G mutant failed to re-sensitize host strains to Trib phage (Fig. 7D). No change in plaque counts was observed between WT RptR induced and repressed conditions for Bam phage, which is resistant to RRDM, indicating that host re-sensitization to Trib phage was not due to a general, RRDM-independent increase in host susceptibility. These data are consistent with our hypothesis that continuous RptR-mediated transcriptional repression is sufficient to silence RRDM anti-phage activity.

### RptR and its binding site are highly conserved across *Gammaproteobacteria*

**Figure 8.**
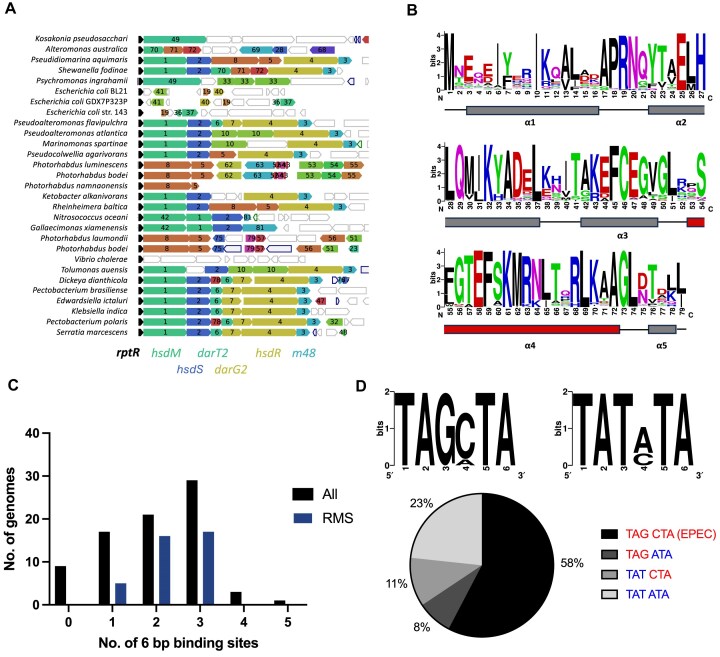
RptR and its 6-bp palindromic binding site are highly conserved. (**A**) Output from Run 1 of gene neighbourhood analysis (webFlaGs) showing conserved genetic association of RptR homologues with Type I RMS (*hsdM*: 1; *hsdR*: 4), DarTG2 (darT2: 6; darG2: 7), and M48 [3] in non-EPEC bacterial genomes (*n* = 29). (**B**) Sequence logo (webLogo) showing conserved amino acid residues across RptR homologues (*n* = 87) identified from webFlaGs. Bars denote areas of secondary structure with the DNA recognition helix ${\boldsymbol{\alpha }}$4 highlighted. (**C**) Frequency distribution of RptR promoters containing between zero and five 6-bp RptR-binding palindromes found in either all 80 promoters identified (All) or in promoters of RptR homologues associated with a Type I RMS (RMS). (**D**) Sequence logo showing four variations of the 6-bp palindrome recognized by RptR identified in the promoters of RptR homologues (*n* = 163 from 80 promoters) (upper). Pie chart showing relative proportions of each variation of 6-bp RptR-binding palindromes in promoters of RptR homologues (*n* = 163 from 80 promoters) (lower).

Next, we sought to determine the distribution of RptR homologues among bacteria and their association with Type I RMS and DarTG2. We employed the gene neighbourhood analysis tool, webFlaGs [45], to identify RptR homologues from the RefSeq database [58], based on amino acid similarity, and gene clusters within 10 ORFs of *rptR*. WebFlaGs was run twice independently with the same RptR input query sequence and the resultant output data (Run 1: Fig. 8A; Run 2: Supplementary Fig. S5) collated and trimmed of duplicated homologues giving 117 non-redundant RptR homologues (Supplementary Table S6). Clusters of frequently associated neighbouring genes of *rptR* are listed in Supplementary Tables S7 (Run 1) and S8 (Run 2). Within this dataset, Type I RMS (*hsdM*, *hsdS*, *hsdR*) were the most commonly associated genetic module adjacent to *rptR* homologues identified by webFlaGs (54/117), with a further smaller subset of these containing *darTG2* (12/54) (Fig. 8A and Supplementary Fig. S5).

Interestingly, RptR is also found directly upstream of non-RMS genetic modules of unknown function, most notably a two-gene cluster comprising a putative RNA-binding protein and AAA-like ATPase (Fig. 8A: clusters 5 and 8; Supplementary Fig. S5: clusters 5 and 6). In four genomes (*Pseudidiomarina aquimaris*, *Rheinheimera baltica*,*Aliidiomarina quisquiliarum*, and*Shewanella xiamenensis*; Fig. 8A and Supplementary Fig. S5), this gene pair was embedded between *hsdS* and *hsdR* in an analogous arrangement to *darTG2* in the RRDM defence island, consistent with this site being a potential recombination hotspot and site for defence systems.

We also determined the degree of protein conservation by alignment of 87 non-redundant full-length RptR homologues (79 amino acids) sharing between 44.3% and 91.1% sequence identity to EPEC RptR (Supplementary Table S9). Of note, there was extremely high sequence conservation within helix α4, which is predicted to be responsible for DNA binding, with Glu58 and Lys61 perfectly conserved across the 87 aligned RptR homologues (Fig. 8B). Furthermore, residues Glu25, Leu69, Ala72, and Leu74 involved in RptR dimerization are also conserved in >99% (86/87) of aligned RptR homologues (Fig. 8B). These data, together with our findings with the L74R and S60G RptR mutants, are consistent with the α4 helix playing critical roles in RptR–DNA binding and dimerization.

Given the conservation of predicted DNA-binding residues in RptR homologues, we reasoned that promoters upstream of *rptR* homologues should contain conserved binding sites. Considering the redundancy between positions G5/T5 and C6/A6 in the 9-bp DR, four possible variations of the 6-bp binding site would be expected to allow RptR binding, i.e. TAGCTA, TAGATA, TATCTA, and TATATA. From the dataset of non-redundant RptR homologues identified above, we extracted 80 associated promoter sequences and quantified the number of RptR binding sites in each promoter (Supplementary Table S10). Interestingly, approximately two-thirds of *rptR* promoters analysed (54/80) contain two or more binding sites (Fig. 8C). Promoters of analysed RptR homologues that also have associated Type I RMS genes were found to be more highly enriched in RptR binding sites with 86% of such promoters containing a minimum of two RptR binding sites (33/38) (Fig. 8C). Within this dataset, no genome containing a single RptR binding site was found to encode a complete Type I RMS. Gene neighbourhoods of the five RMS-associated RptR promoters containing a single RptR binding site (*Neiella litorisoli*, *Providencia heimbachae*, *Shewanella baltica*, *Leclercia* sp. LTM14, *Litoreibacter janthinus*; Supplementary Fig. S5) were found to lack the *hsdS* gene encoding the Type I RMS specificity subunit. These data are consistent with our model in which RptR repression of a functional Type I RMS is contingent on the presence of at least two binding sites.

To investigate the relative frequencies of the four RptR binding sites, we next quantified the number of each of the 6-bp binding site variants in each of the 80 promoters identified. Seventy-one *rptR* promoters from our dataset were found to contain at least one RptR binding site, with the EPEC sequence (TAGCTA) representing 58% of all predicted RptR binding sites (Fig. 8D). In binding sites with a 5′ TAT half-site, there is a slight preference for adenine at position 6 (seen in 67% of sites), with the remainder containing C6 (Fig. 8D). There were no 6-bp RptR binding sites containing guanine at position 4, consistent with RptR failing to bind to a TAGGTA motif.

Within the identified set of *rptR* promoters containing two binding sites, 9 out of 21 were found to contain a non-binding 6-bp sequence flanked by two predicted functional 6-bp RptR binding sites, an arrangement reminiscent of DR^1/s/3^. Given that RptR binds to DR^1/s/3^ promoter sequences at full occupancy resulting in WT-like gene repression, we predict that these promoters will be repressed by RptR. These findings lend further support to the model that two RptR binding sites, whether adjacent or separated by an intervening sequence, are necessary and sufficient for RptR binding and repression of genes contained in defence islands.

## Discussion

**Figure 9.**
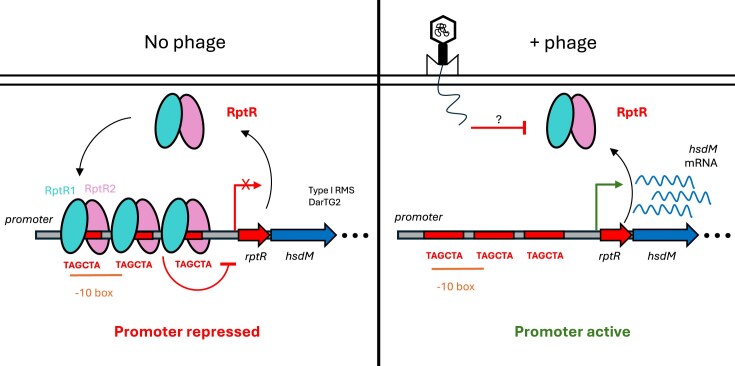
Summary model of RptR-mediated transcriptional repression.

Here, we show that the RRDM island provides *E. coli* with robust defence against several lytic phages from the Durham collection [34]. This island contains DarTG2 embedded within a Type I RMS between *hsdS* and *hsdR*. We demonstrate that the first gene of RRDM island encodes a 9-kDa transcriptional repressor, RptR, which regulates its own promoter, and present the crystal structure of an RptR homodimer. We propose that, in the absence of phage infection, RptR binds to a series of three 6-bp palindromic repeats that overlap with the −10 box, leading to transcriptional repression. Upon phage infection, we observe upregulation of *hsdM* that we hypothesize to be elicited through phage-induced removal of RptR through an unknown mechanism (Fig. 9). Both the DNA binding site and in particular the α4 helix of RptR are highly conserved in potential defence islands in diverse bacterial species, indicating that EPEC RptR is an exemplar of a widespread mode of defence island regulation.

Intriguingly, initial bioinformatic analyses of RptR failed to identify any homologues in the PDB. Bacterial transcriptional regulators typically contain a sensor domain and a DNA-binding domain enabling effective coupling of incoming signals to transcriptional control. RptR, instead, is a single domain protein consisting of four alpha helices that form a remarkably compact repressor. To our knowledge, RptR is one of the smallest DNA-binding regulators in *E. coli*. The 12-kDa CedA transcription factor, which controls cell division, bridges distant domains of RNA polymerase holoenzyme but does not bind DNA directly [59].

In contrast, phage transcriptional regulators, such as the lambda repressor protein cI, are often substantially smaller than their bacterial counterparts. cI contains a HTH motif that shares superficial similarities with RptR; in both cases, a DNA recognition helix is positioned at a right angle to a third helix within a tri-helical bundle [52]. cI also binds to DNA as a homodimer with each monomer inserting an alpha helix into the major groove of DNA [53]. However, while the cI repressor and other HTH regulators typically engage two adjacent major grooves spanning a 15–20-bp palindrome, AlphaFold predictions, EMSAs, and *in vivo* analyses indicate that RptR binds as a dimer to opposite faces of a single major groove [52–54]. This accounts for the short 6-bp palindrome that we show binds RptR.

Short (<8 bp) palindromic binding sites are a hallmark of the eukaryotic basic leucine zipper (bZIP) transcription factors that form a homo- or heterodimer comprising two splayed alpha helices that interact with opposite faces of a single DNA major groove [60], analogous to RptR. Although RptR lacks the extensive C-terminal Leu-rich dimerization domain of bZIP transcription factors, the C-terminal dimerization interface of RptR contains four highly conserved leucine residues spaced three or four amino acids apart. bZIP transcription factors were thought to have arisen in the last common ancestor of eukaryotes [60]. However, a *Pseudomonas putida* leucine zipper protein, TodS, has been described that binds a 6-bp palindrome in its native promoter [61]. We speculate that RptR represents an even more primitive bZIP-like transcriptional regulator that lacks the extended C-terminal dimerization domain of the eukaryotic regulators even though it shares structural and DNA-binding characteristics with bZIP regulators. Investigation into the evolutionary relationships of RptR with TodS and other more distant eukaryotic bZIP proteins should provide intriguing insights into the evolution of this widespread class of transcriptional regulators.

Despite the presence of three RptR binding sites in the EPEC RRDM promoter, we found that only two binding sites are needed for RptR binding and promoter repression. We propose that the presence of an additional binding site effectively increases the sensitivity of the promoter to repression at low RptR concentrations. The converse has been described for transcriptional activators where increasing the number of binding sites leads to increased gene expression [62]. The need for sensitive transcriptional repression of a defence island is not obvious as the toxic components of Type I RMS and DarTG systems are usually counteracted by their cognate methyltransferase and antitoxin, respectively. In addition, regulation of Type I RMS activity can also occur post-translationally. For instance, the endonuclease HsdR subunit of EcoKI is degraded by the ClpXP protease to enable the RMS methyltransferase complex to modify unmethylated DNA in a new host, in a process termed restriction alleviation [26, 63]. We hypothesize that RptR offers a form of defence island regulation that negates the need for continuous protein turnover and acts as an additional layer of regulation akin to the transcriptional C proteins of Type II RMS. In contrast to RptR, C proteins are typically encoded upstream of the restriction endonuclease and bear DNA-binding and structural similarities with the lambda cI HTH transcriptional repressor [29, 30, 64].

An unresolved question is how RptR controls the response to phage infection. Transcript levels of *hsdM*, immediately downstream of *rptR*, are markedly upregulated upon phage infection, indicating relief of RptR-mediated repression. However, in the first instance, upregulation of the RRDM promoter should increase RptR levels, which would reduce expression of genes on the island in a negative feedback loop. Therefore, it seems likely that activation occurs through either proteolytic degradation or physical sequestration of RptR by phage and/or host factors. The transcriptional repressor of some CBASS systems, CapH, is de-repressed through proteolytic degradation by its cognate protease, CapP [33]. Meanwhile, the antitoxin domain of CapRel is blocked via physical binding of phage capsid proteins [24]. Given general host transcription and translation are commonly shut off during early phage infection and levels of any sequestrating binding partner must titrate out increasing levels of RptR, any such binding partner is likely to be phage derived. Further studies are underway to determine the mode of RptR de-repression and identify potential phage-encoded RptR interacting proteins.

In summary, RptR is a small, highly conserved regulator of Type I RMS that is widely distributed in defence islands across *Gammaproteobacteria*. Conservation is not restricted to RptR but also includes its DNA binding motif. Preservation of the sequence of the regulator and its target sequence in a diverse range of bacteria highlights its functional effectiveness in regulating defence against phage infection. An improved understanding of how bacteria modulate their anti-phage defence systems could not only provide insights into phage–host interactions but could also offer novel strategies for eliminating bacterial pathogens, many of which harbour multiple defence systems that could be activated in the absence of phage to kill their bacterial host.

## Supplementary Material

gkaf645_Supplemental_Files

## Data Availability

The raw data underlying this article will be shared on reasonable request to the corresponding author. X-ray crystallography data for the molecular structure of RptR have been deposited at the Protein Data Bank (PDB: 9R2Z).
