# Supplementary material for: Regulation of a phage defence island by RptR, a novel repressor that controls restriction–modification systems in diverse bacteria
Source: Nucleic Acids Res. 2025 Jul 12;53(13):gkaf645. doi: 10.1093/nar/gkaf645 (PMC12255303; doi:10.1093/nar/gkaf645)
Supplement: gkaf645_Supplemental_Files [file gkaf645_supplemental_files.zip › RptR_paper_supplementary_figures_REV_FINAL_2.pdf]

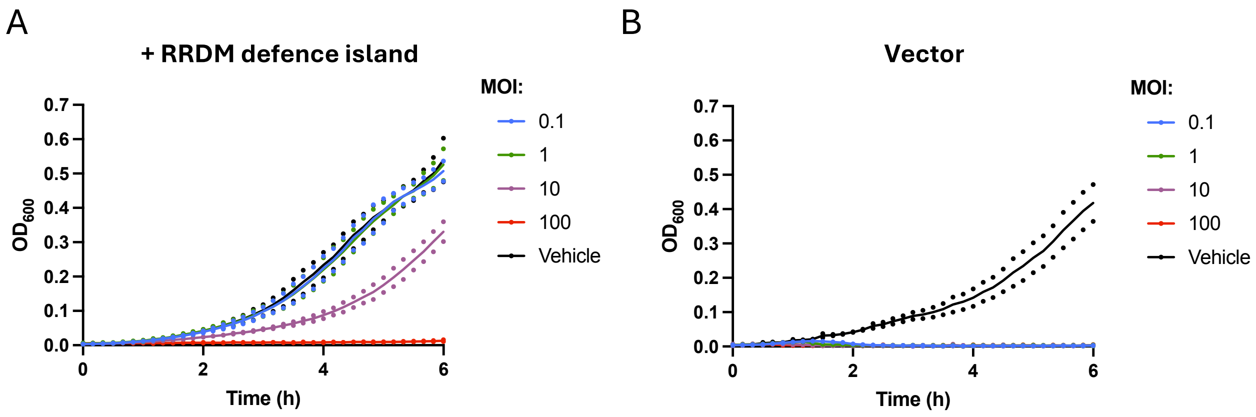

Supplementary Figure 1 – Trib phage infection timecourse

A-B: *E. coli* DH5α strains carrying pBR322-RRDM (**A**) or empty pBR322 (**B**) vectors were infected with Trib phage at the indicated multiplicities of infection (MOI), or a vehicle (phage buffer) control. Cell density, taken as absorbance at 600 nm (OD600), was measured at intervals of 10 minutes post-infection. Lines represent mean of n = 2 biological replicates with values for individual replicates shown.

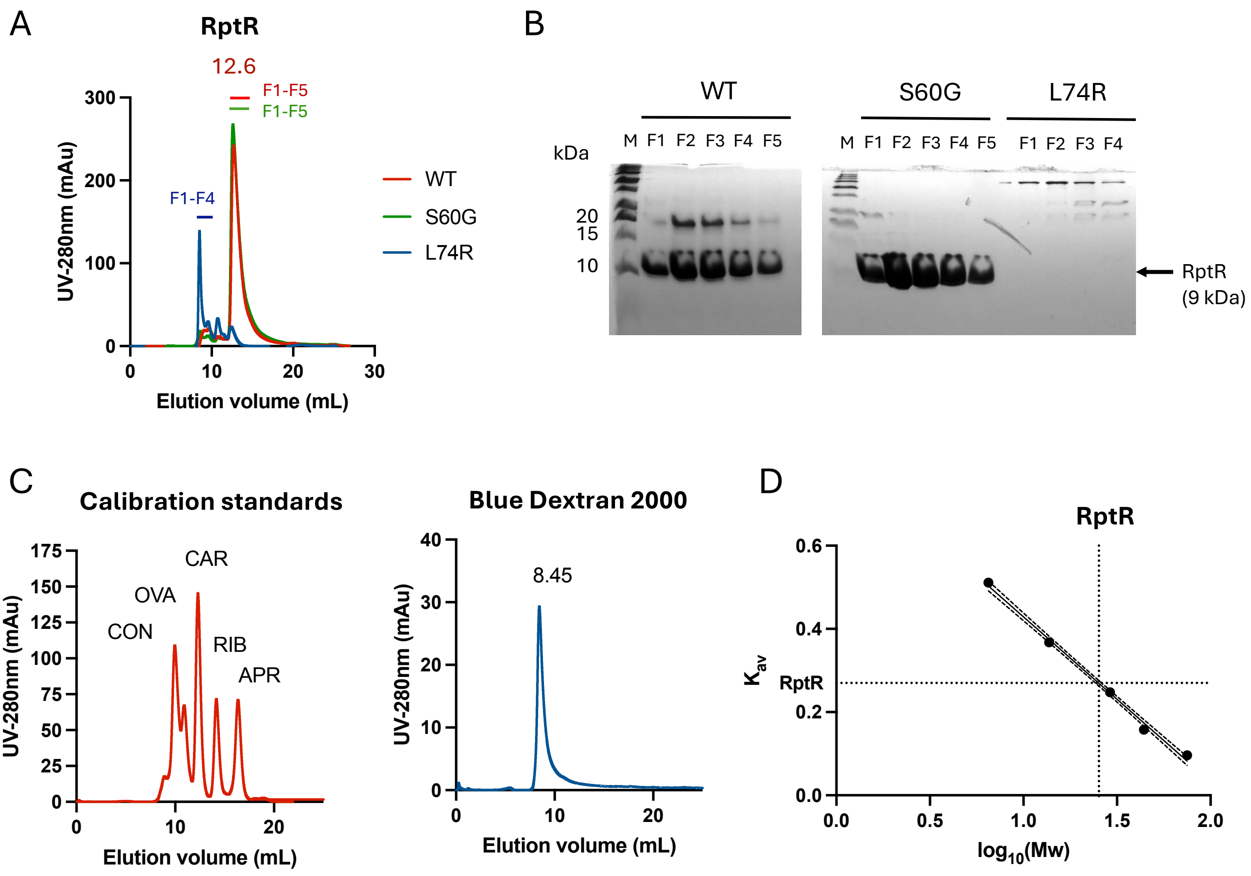

Supplementary Figure 2 – Analytical SEC of RptR

A. Size exclusion chromatogram of WT RptR and RptR L74R and S60G mutants. Values represent protein absorbance (mAu, 280nm) over one column volume (Superdex S75 increase 10/300 GL). Horizontal lines above peaks denote volumes of elution fractions collected for SDS-PAGE analysis. **B.** Coomassie stained SDS-PAGE gels of size-exclusion chromatography (SEC) elution fractions of WT (F1-F5), S60G (F1-F5), and L74R RptR (F1-F4) and molecular weight marker (M). Sizes of molecular weight markers in kilodaltons (kDa) are shown. **C.** Size exclusion chromatogram of molecular weight standards (CON: conalbumin, 75 kDa; OVA: ovalbumin, 44 kDa; CAR: carbonic anhydrase, 29 kDa; RIB: ribonuclease A, 14 kDa; APR: aprotonin, 7 kDa) (left), Blue Dextran 2000 (middle), with void volume (V_o, mL) indicated, and RptR (right), with elution volume indicated, run on a Superdex S75 increase 10/300 GL column. **D.** Standard curve of K_{av} (($V_e - V_o$)/($V_c - V_o$), where V_e represents elution volume (mL) and V_c represented total column volume (mL) against the base 10 logarithm of the molecular weight (kDa) ($\log_{10}$(MW)) of the standards used in (A). Values represent the mean K_{av} values for each standard in (A) with SD (shaded) of n = 3 independent experiments. K_{av} of RptR is indicated by the dotted line (y-axis) and the interpolated $\log_{10}$(MW) indicated on the x-axis.

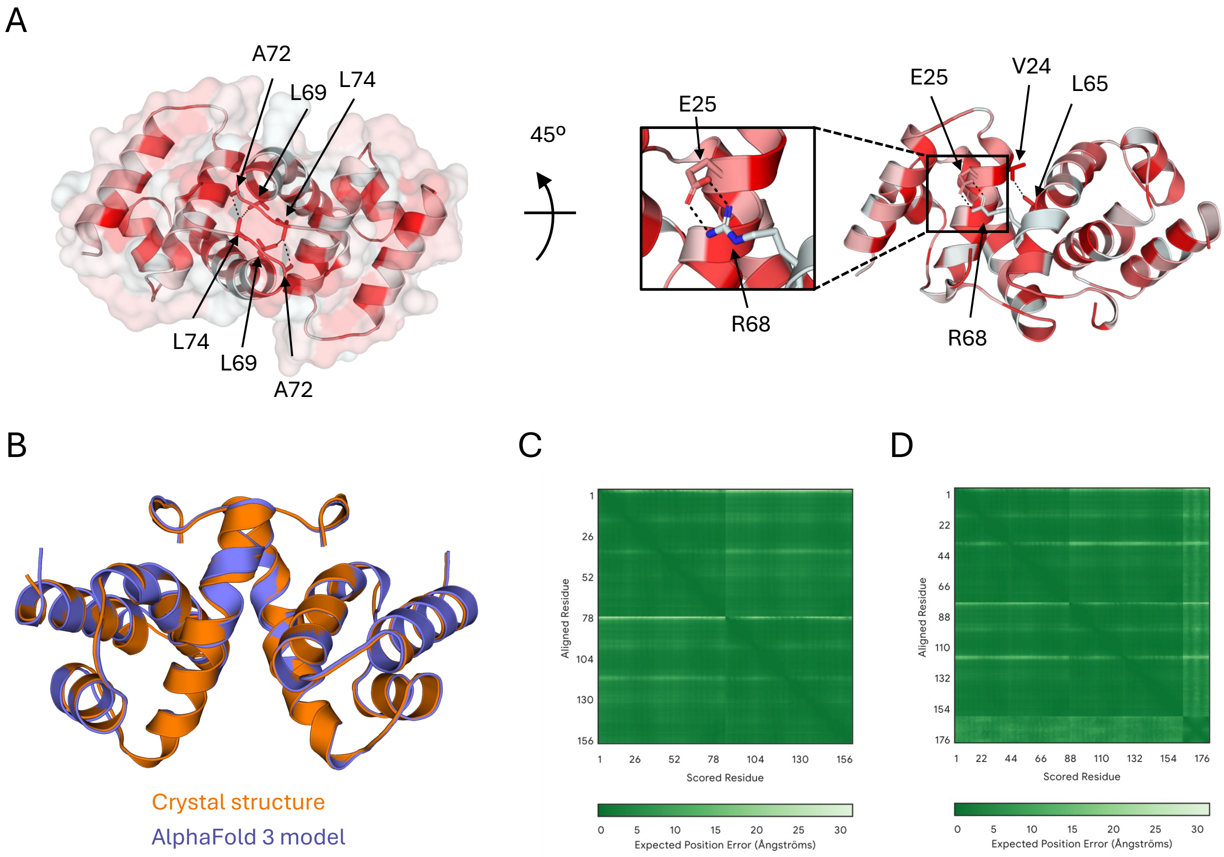

Supplementary Figure 3 – Dimerisation interface of RptR and AlphaFold 3 models of RptR (apo and DNA-bound)

A. Dimerisation interface from crystal structure of RptR colored according to the Eisenberg hydrophobicity scale (41). Residues predicted to form part of the dimerisation interface are shown as sticks (V24, E25, L65, R68, L69, A72, L74). Close-up view of the electrostatic interaction between Glu25 and Arg68 with residue sticks colored according to chemical element (O, red; N, blue). **B.** Superimposition of crystal structure of RptR with the AlphaFold 3 predicted model of dimeric RptR (RMSD = 0.503 Å). **C.** Predicted aligned error (PAE) plot for the AlphaFold 3 predicted structure of dimeric RptR without DNA (ipTM: 0.89, pTM: 0.9). **D.** PAE plot for the AlphaFold 3 predicted structure of dimeric RptR with 9 bp IR (dsDNA sequence: TTTAGCTAA) (ipTM: 0.9, pTM: 0.92).

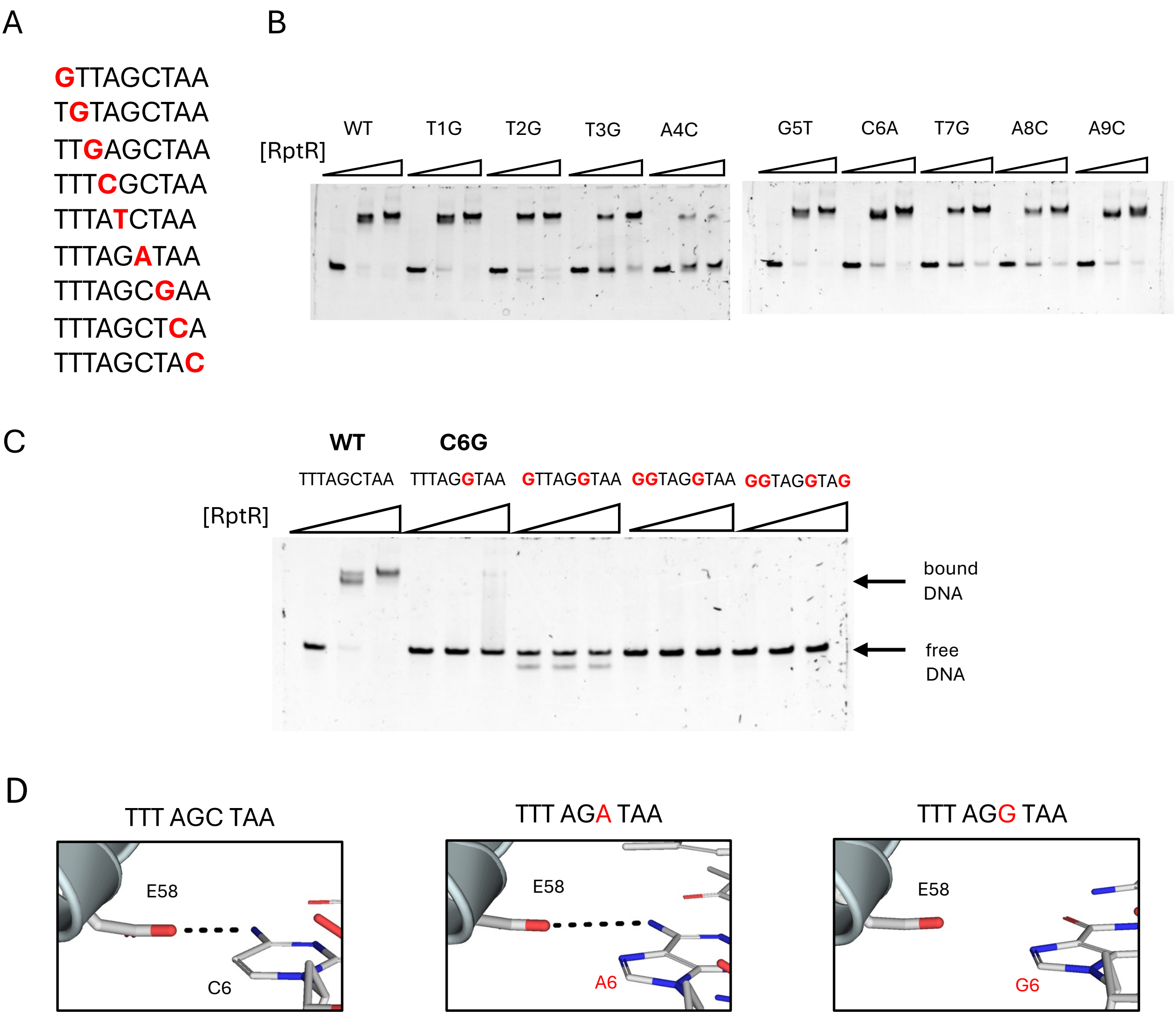

Supplementary Figure 4 – RtpR binding is abolished with a C5G mutation introduced on a 3x IR dsDNA probe

A. Mutational key used for introduction of point mutations into each IR in dsDNA probes. Red bases indicate mutated positions where thymine-guanine or adenine-cytosine (and vice versa) transversions have been introduced. **B.** EMSA showing binding of His_6-3C-RptR to dsDNA probes containing three IRs (3x IR) with the indicated point mutations. Point mutations were introduced according to the mutational key in (A). Image representative of n = 2 replicates. **C.** EMSA showing binding of His_6-3C-RptR to unlabelled 60 bp dsDNA probes containing three 9 bp IRs with any mutated bases highlighted in red. Purified protein (0, 0.6, 1.2 μM) was incubated with dsDNA probes (0.05 μM) for 30 min at 37°C prior to visualisation on an 8% TBE-PAGE gel. Image representative of n = 2 replicates. **D.** Predicted interaction between RptR Glu58 (E58) and cytosine (C6, wild-type, left), adenine (A6, center) or guanine (G6, right) at position 6 of one IR.

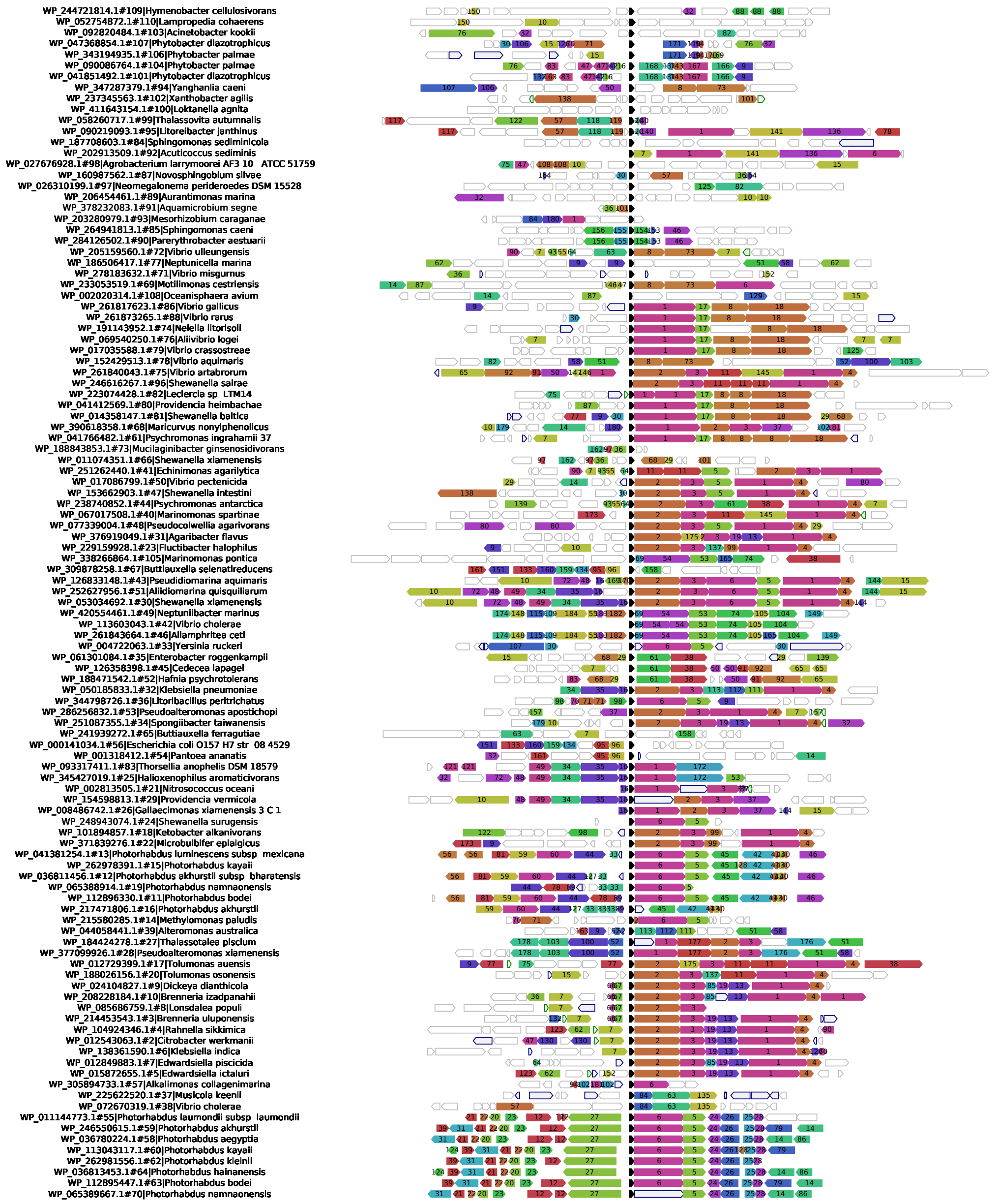

Supplementary Figure 5 – Gene neighbourhood analysis of *rptR*

Output from Run 2 of gene neighbourhood analysis (webFlaGs) showing genetic association of RptR homologues with Type I RMS (*hsdM:* 2; *hsdS:* 3; *hsdR:* 1), DarTG2 (*darT2:* 19; *darG2:* 13) and M48 (4) in non-EPEC bacterial genomes (n = 109).
